# Development of Recombinase Polymerase Amplification and CRISPR-Cas12a–Enhanced Isothermal Amplification Assays for *Strongyloides stercoralis* DNA Detection: A Pilot Study

**DOI:** 10.4269/ajtmh.26-0042

**Published:** 2026-04-21

**Authors:** Robertine Lontuo-Fogang, Sasisekhar Bennuru, Thomas B. Nutman

**Affiliations:** Laboratory of Parasitic Diseases, National Institute of Allergy and Infectious Diseases, National Institutes of Health, Bethesda, Maryland, USA

## Abstract

Soil-transmitted helminth (STH) infections are prevalent worldwide, but the true burden of strongyloidiasis is unclear due to lack of sensitive and field-friendly diagnostic tools. Diagnosis is often based on serological assays that are typically not point-of-care (POC). Although polymerase chain reaction (PCR) tests are sensitive and specific, the need for expensive equipment and highly skilled personnel limits their use in resource limited areas. Isothermal amplification assays are largely instrument-free, making them simpler to implement without loss of either sensitivity or specificity. We developed two recombinase polymerase amplification (RPA) assays to detect *Strongyloides stercoralis* (*Ss*) in human stool samples and a complementary CRISPR-Cas12a detection system with visual readouts. Primers, probes, and guide RNAs (crRNAs) for these assays were designed targeting the *Ss*-NIE sequence and *Ss* dispersed repetitive sequence (*Ss*-DRS). The assay’s specificities and limits of detection (LOD) were assessed using gDNA from *Ss* L3 larvae or from other STH and filariae. The NIE RPA showed a LOD of 1 fg/*µ*L, whereas the LOD for the *Ss*-DRS RPA was 1 pg/*µ*L. The LOD was 500 fg/*µ*L for the NIE RPA CRISPR-Cas12a assay. No cross-reactivity with any filarial parasite or other STH was observed. Because the NIE assays were more sensitive than the *Ss*-DRS assay, six patient samples positive for *Ss* by real-time PCR (qPCR) were tested using the NIE assays, of which four were positive. Though assay refinement and clinical validation are needed, this study establishes fast, highly sensitive and field-applicable POC diagnostic tools for *Ss* detection that are ideal for use in endemic areas with limited resources.

## INTRODUCTION

*Strongyloides stercoralis* (*Ss*) is a soil-transmitted helminth (STH) that infects millions of individuals globally. Although infections are typically subclinical or present with mild clinical manifestations, *S. stercoralis* possesses a unique autoinfective life cycle that enables chronic, potentially lifelong persistence within their host^1^^–^^3^ and the potential for driving hyperinfection syndromes with mortality associated with dissemination that can range from 15% to 87%.^4^ The global prevalence is estimated to be 613.9 million individuals, with the highest burden occurring in tropical and subtropical regions in resource-limited communities where sanitation and hygiene are often problematic.^5^ Despite its clinical importance, *Ss* remains one of the most neglected parasitic infections, largely due to diagnostic challenges and limited surveillance capacity.^2^ Thus, there is a need to develop diagnostic tools that can be used at point-of-care (POC) and for evaluating public health burden.

The standard of diagnosis for *Ss* remains multiple stool examinations (usually over days) for detection of larvae using time-intensive methodologies such as direct microscopy, Baermann concentration and agar plate culture, which are relatively insensitive due to low parasite burdens and intermittent release of larvae from upper intestine-located adult worms.^6^^–^^8^ Serological assays (usually ELISA), based on the detection of IgG or IgG4 antibodies to either crude somatic *S. stercoralis* (or *S. ratti*) antigen or to the *Ss*-specific recombinant antigens NIE or *Ss*IR,^6^^,^^9^^–^^14^ have been useful, but cross-reactivity with other helminths,^15^^,^^16^ their failure to disappear in a timely manner post treatment,^9^^,^^10^^,^^13^ and the need for specialized equipment and personnel make it difficult to be useful for specific diagnosis in highly *Ss*-endemic areas.

Molecular diagnostic tools (e.g., polymerase chain reaction [PCR]) have high sensitivities and specificities. Previously, real-time PCRs (qPCRs) targeting highly repetitive tandem sequences were developed with very high analytical sensitivity and specificity in *Ss* and other soil transmitted helminths.^17^^–^^20^ However, the cost, the instrumentation, and the need for highly trained personnel place this largely out of reach for all but large centralized laboratories. Advances in isothermal amplification technologies such as loop-mediated isothermal amplification (LAMP), recombinase polymerase amplification (RPA), and clustered regularly interspaced short palindromic repeats (CRISPR)–based nucleic acid detection platforms have enabled rapid, field-deployable, and less expensive molecular diagnostics. Watts et al.^21^^,^^22^ developed a LAMP assay targeting the 28S ribosomal subunit gene to detect *Ss* in different specimen matrices, including bronchoalveolar lavage fluid, serum, and stool. Because RPA operates at low constant temperatures (25–42°C) and can generate detectable amplicons within 15–30 minutes, it would be suitable for decentralized testing and POC applications.^10^^,^^23^ CRISPR-Cas12a detection systems can complement isothermal amplification by providing additional sequence-specific recognition and collateral reporter cleavage, enabling highly specific readouts with fluorescence or lateral-flow formats. Combining these technologies has demonstrated promising results for pathogen detection in a range of parasitic and viral diseases,^24^^,^^25^ but relatively few studies have explored their application to strongyloidiasis. Recently, a RPA-lateral flow targeting the *Ss* 18S rRNA gene in human stool reported analytical LOD of 20 pg/*µ*L, a sensitivity of 77.2% and a specificity of 73.1% using qPCR as gold standard.^23^ We developed two different RPAs targeting either the *Ss*-NIE gene family or *Ss*-DRS that have diagnostic promise as a POC tool for *Ss*.

## MATERIALS AND METHODS

### Patients and sample collection.

All stool samples involved in this study were collected as part of several National Institutes of Health (NIH) Intramural Institutional Review Board–approved studies performed under the auspices of the Laboratory of Parasitic Diseases, National Institute of Allergy and Infectious Diseases (NIAID) (NCT00001645, NCT00001230). All patients or parent/guardian of patients provided informed written consent.

### Primer/probe design and screening.

**Figure 1.**
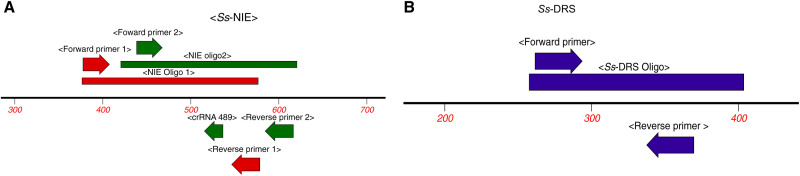
Design strategy of selected primers/probes for EXO RPA (red) and RPA–CRISPR-Cas12a (green) assays based on the NIE antigen sequence (**A**), and EXO RPA based on the dispersed repetitive sequence in blue (B).

Figure 1 shows primers and probes design strategy for the two *Ss*-specific molecular targets–the immunodiagnostic antigen NIE sequence and the *Ss*-DRS–to exploit high copy number tandem repeats. Primers and probes for both the exonuclease (EXO) and basic RPA assays were designed with PrimedRPA (https://github.com/MatthewHiggins2017/bioconda-PrimedRPA),^26^ with parameters set for at least 40% guanine-cytosine (GC) content, primer length, and probes ranging from 28–32 and 45–50 base pairs, respectively. The maximum amplicon length was set at 300 and also checked against background cross-reactivity with other helminths. These primers were then analyzed using the Oligo Analyzer^™^ (IDT, Coralville, IA) tool to check for the presence of primer dimers and secondary structure formation, among other parameters. Promising primer sets were further analyzed using the National Center for Biotechnology Information (NCBI) BLAST tool to check for additional cross-reactivity (http://blast.ncbi.nlm.nih.gov/Blast.cgi). Due to the A-T richness of both sequences, only a single potential forward primer and probe were identified for the NIE EXO RPA. Two forward primers each were identified for the *Ss*-DRS EXO and the NIE basic RPAs. These forward primers were paired with multiple reverse primers. For the EXO RPAs, a total of seven primer/probe sets were processed—four targeting the *Ss*-DRS gene (accession number AY028262.1) and three targeting the *Ss*-NIE antigen sequence (accession number (AF136445.1), whereas only two primers targeting the *Ss*-NIE sequence were designed for the basic RPA (Supplemental Table 1). All the probes were labeled with a 6FAM fluorophore at the 5′ end and quenched with the Iowa black hole quencher (BHQ) at the 3′ end (IDT).

For the RPA CRISPR-Cas12a assay, a generic double-quenched, single-stranded DNA reporter probe (5′-/56-FAM/TTA TT/3IABkFQ/-3′) (IDT) was used. LbCas12a guide RNAs (crRNAs) were designed targeting the *Ss*-NIE sequence using MacVector (v. 18.7.6; MacVector, Inc., Apex, NC). crRNAs that fell within the basic RPA amplicon range without any overlap with the RPA primers were selected for this purpose (Supplemental Table 2). Synthetic oligos (200 bp) were also generated for each target and used as positive controls (see Supplementary data). Overlapping oligos were generated for *Ss*-NIE due to size limitations. Nuclease-free water was used as no template control (NTC) in each experiment.

### Preparation of RPA reactions.

The RPA reactions were performed in a 50 *µ*L total volume by using the TwistAmp EXO and Basic lyophilized kits (TwistDX, Cambridge, United Kingdom), respectively, for the RPA and RPA–CRISPR-Cas12a assays. All assays were run using the basic kit without the probe and checked by gel electrophoresis to ensure the expected size. For the basic assay, a master mix (46.5 *µ*L) comprising 2.1 *µ*L of 10 *µ*M forward and reverse primers each, 29.5 *µ*L of primer free rehydration buffer, 12.8 *µ*L of nuclease-free water was added to the TwistAmp lyophilized pellet and mixed by pipetting until pellet was completely dissolved. One microliter of the template was then added, and a total of 2.5 *µ*L of 280 nM magnesium acetate added. The tubes were vigorously mixed and immediately incubated at 40°C for 30 minutes using the Applied Biosystems^™^ Veriti^™^ Thermal Cycler. The procedure was identical for the EXO assays using the same primers except that 0.6 *µ*L of 10 *µ*M probe was added to the reaction.

### RPA–CRISPR-Cas12a detection.

The CRISPR-Cas12a reactions were prepared in a master mix (15 *µ*L) containing 1.5 *µ*L of 10 × NEBuffer r2.1, 0.5 *µ*L of 1 *µ*M LbaCas12a enzyme, 1.5 *µ*L of 1 *µ*M crRNA, and 10 *µ*L PCR-quality water. The master mix was incubated at room temperature for 20 minutes to generate crRNA-Cas12 complexes. Ten microliters of 10 *µ*M probe were then added to the reaction. This was mixed by pipetting, then 14 *µ*L of the mixture aliquoted into a 96-well PCR plate and 1 *µ*L of the basic RPA product added. This was then incubated at 37°C for 40–60 minutes. For all assays, *S. stercoralis* gDNA was tested at concentrations of 100 pg/*µ*L, 1 pg/*µ*L, 100 fg/*µ*L, 1 fg/*µ*L, 100 ag/*µ*L, and 1 ag/*µ*L. PCR-quality water was used as no template control and the *Ss* oligos designed for each assay as positive controls.

### Readout methods and data analyses.

Visualization of the basic RPA amplicons was done with the Bioanalyzer 2100 (Agilent Technologies, Santa Clara, CA) using a 1500 bp DNA ladder to determine the predicted amplicon size. For each assay, fluorescent signal was monitored using the QuantStudio ViiA 7 Pro Real-Time PCR System (QuantStudio^™^, Thermo Fisher Scientific, Waltham, MA) and visually under a blue light transilluminator. Fluorescent intensity was quantified using the open-source image processing software, ImageJ (https://imagej.net/ij/download.html). Cutoff for positivity was determined by multiplying the mean fluorescence intensity of the negative control by 1.5 in each experiment. Anything above the obtained value was considered positive. All data analyses were performed with Prism v. 10.4.1 (GraphPad, Inc., Boston, MA) with two-sided *P* <0.05 level of significance.

## RESULTS

### RPA primers/probes validation and optimization.

**Figure 2.**
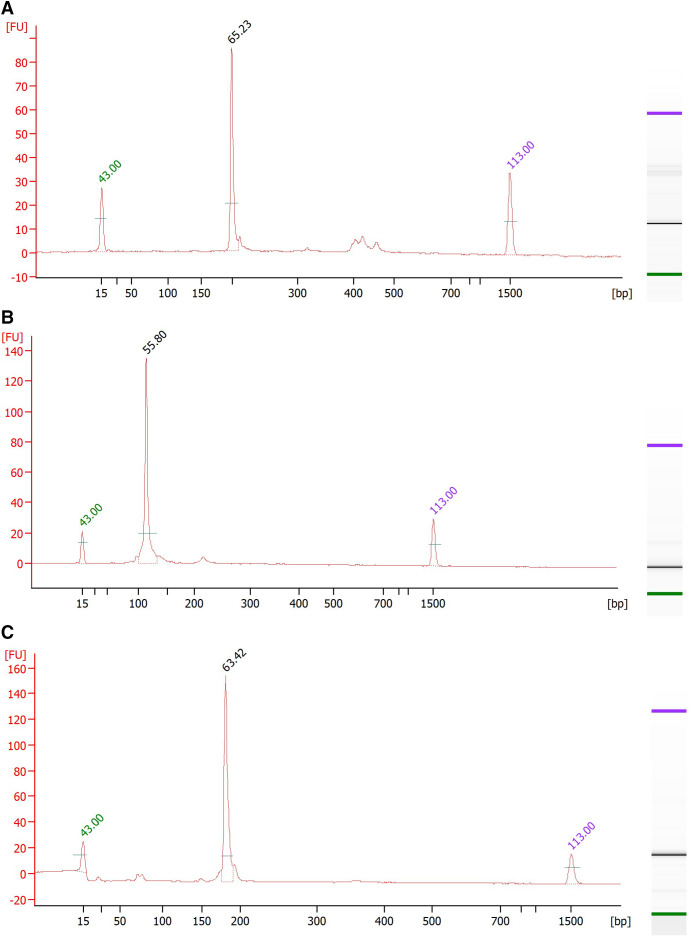
Electrophoresis characteristics of selected primer sets. Visualization of RPA products tested on gDNA show exclusive bands at (**A**) 200 bp for NIE RPA, (**B**) 118 bp for *Ss*-DRS RPA, and (**C**)180 bp for NIE CRISPR-Cas12a assays.

After testing each forward primer in combination with their reverse on synthetic oligos and gDNA, promising primer probe sets were chosen and tested at different reaction temperatures and incubation times. One set was picked from each assay for further optimization. As can be seen on the gel electropherogram image in Figure 2, visualization of the RPA products from each assay revealed amplicons at the expected size of 200 bp for the NIE EXO RPA, 118 for the *Ss*-DRS EXO RPA, and 180 for the NIE CRISPR-Cas12a assay. Optimal amplification was achieved at 40°C in just 30 minutes for the EXO RPAs, whereas the CRISPR-Cas12a assay was at 37°C for 1 hour. Table 1 shows the selected primers and probe sequences after optimization. After testing and optimizing 4 different crRNAs, the crRNA 489 (TTTA-AAATTATATAAAGCTATTTCA) was chosen for the CRISPR-Cas12a system.

**Table 1:** RPA and RPA–CRISPR-Cas12a primer/probe sequences selected for each assay

Assay Design	Target	Forward Primer	Probe	Reverse Primer
EXO RPA	*Ss*-NIE	5′GCTCAAGCTTATGCTG AAGTAATTGCTAG-3′	5′GAGGGAACAGGAGAAAATCT TGCTTATGGAACAACA/iFluorT/ T/idSp/AT/iBHQ-1dT/GGC CA 3′	5′TGTGTAAAATGTCCAGTT GCAGGACTAAATCC-3′
EXO RPA	*Ss*-DRS	5′ACGCTCCAGAATTAGTT CCAGTTGAATAACAG-3′	5′AACAGTCTCCAGTTCACTCCAG AAGAG/iFluorT/T/idSp/C/iBHQ- 1dT/ATAATCCTAACTC-3′	5′TTGTGAGGGGTTGGAAA CTGTTGCTTTACTGG-3′
RPA–CRISPR-Cas12a	*Ss*-NIE	5′CGAATTGAGGGAACAGG AGAAAATCTTGC-3′	N/A	5′CACCAAATCCAGCATGA GTTGTACCTTTCCAT-3′

CRISPR-Cas12a = clustered regularly interspaced short palindromic repeats - Cas12a enzyme; EXO RPA = exonuclease recombinase polymerase amplification; N/A = not applicable; *Ss*-DRS = strongyloides stercoralis dispersed repetitive sequence; *Ss*-NIE = strongyloides stercoralis NIE sequence.

### Determination of limit of detection of EXO RPA assays and NIE RPA–CRISPR-Cas12a on stored gDNA extracted from *Ss* larvae.

**Figure 3.**
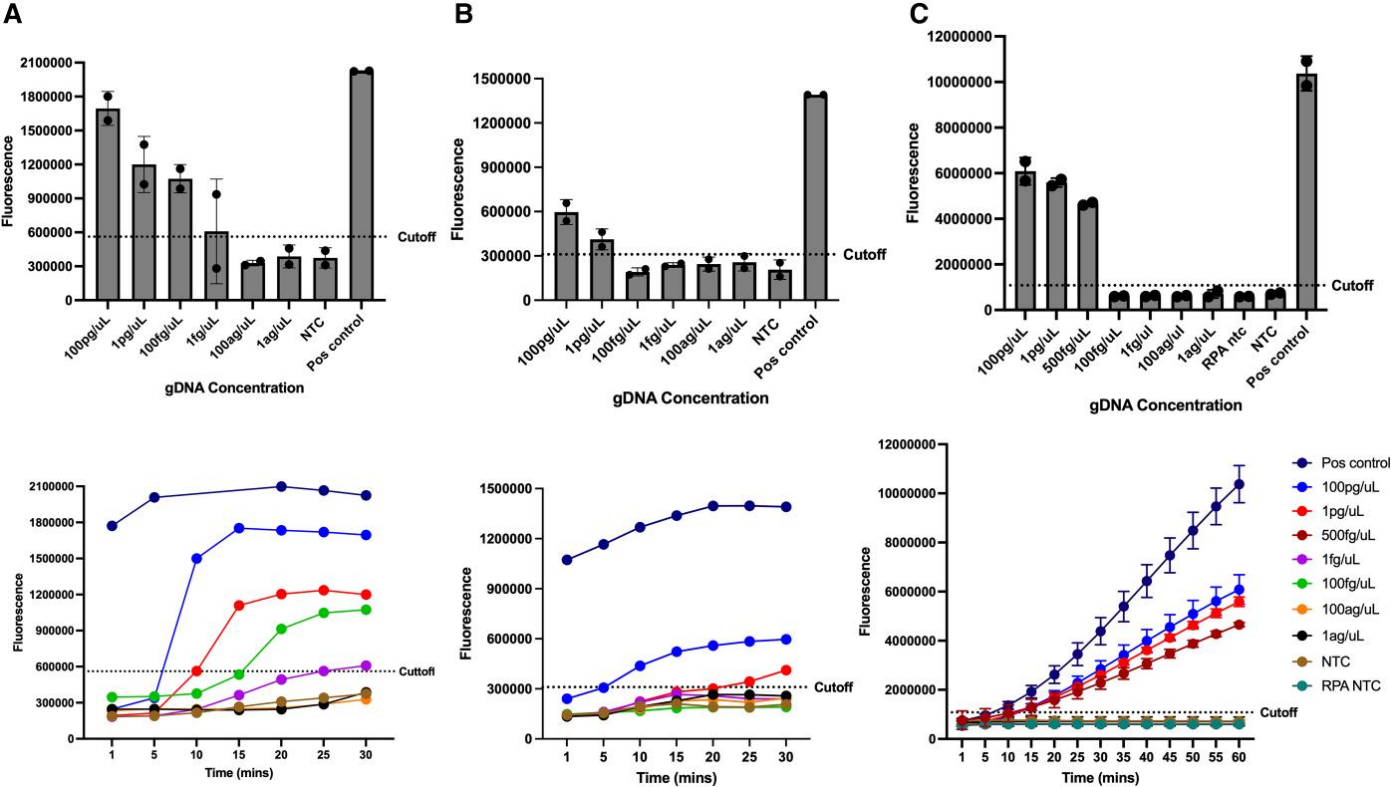
RPA and RPA-Cas12a assays limits of detection showing reaction development with time on the bottom and their respective endpoints (30 minutes) on the top row. (**A**) NIE EXO RPA with 1 fg/*µ*L LOD; (**B**) *Ss*-DRS EXO RPA with 1 pg/*µ*L LOD, and (**C**) NIE RPA-Cas12a showing LOD of 500 fg/*µ*L.

Figure 3 shows the analytical sensitivity of each assay using 100-fold serial dilutions of *Ss* gDNA. The NIE EXO RPA assay achieved a limit of detection (LOD) of 1 fg/*µ*L, whereas the *Ss*-DRS EXO RPA demonstrated a sensitivity of 1pg/*µ*L. The CRISPR-Cas12a assay targeting NIE produced detectable collateral cleavage fluorescence to 500 fg/*µ*L. In addition, half reactions were also performed for the NIE EXO RPA assay for the purpose of minimizing reagents, and LOD only reached 100 fg/*µ*L, which is still very satisfactory (Supplemental Figure 1).

### Measurement of fluorescence intensity.

**Figure 4.**
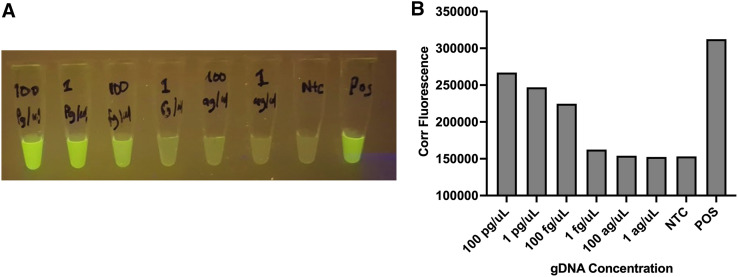
Representative image of fluorescence intensity. Visual readouts under blue light transilluminator shows diminishing fluorescence as concentrations of *Ss* gDNA decreases (**A**) and corrected fluorescence measured with Image J after subtracting background noise (**B**). Figure 4A image credit: Robertine Lontuo-Fogang.

Fluorescence of the RPA reactions could be visually and qualitatively read using a blue light (and orange filter) as seen in Figure 4A. This fluorescence could be further quantified using ImageJ where pixel density quantified the reduction in fluorescence intensity with decreasing gDNA concentrations (Figure 4B).

### Species specificity of assays.

**Figure 5.**
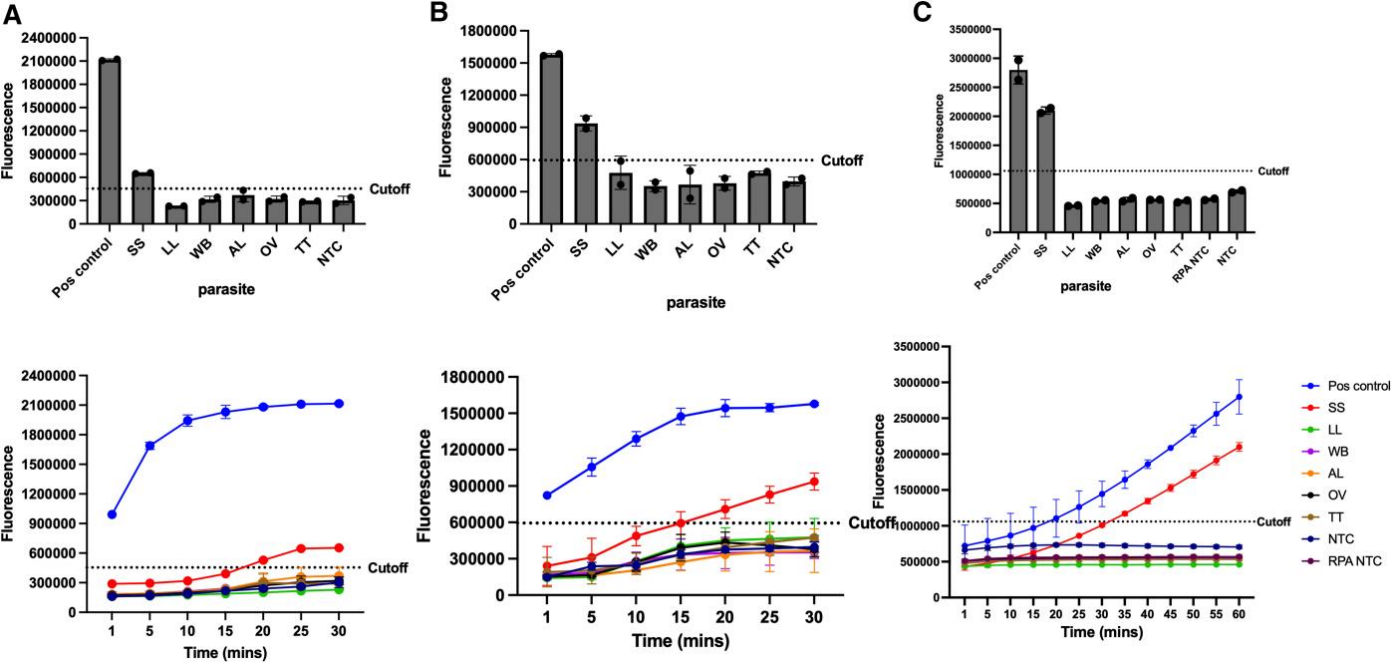
Specificity showing no amplification with other helminth parasites for all three assays. The top row shows reaction endpoints and the time course on the bottom. (**A**) NIE EXO RPA with 1 fg/*µ*L *Ss* gDNA. (**B**) *Ss*-DRS EXO RPA with 100 pg/*µ*L *Ss* gDNA. (**C**) NIE RPA-Cas12a with 100 pg/*µ*L *Ss* gDNA. Higher *Ss* gDNA concentration was used on **B** and **C** due to their lower LODs. All other parasites were tested at 1 pg/*µ*L gDNA.

To ensure the species specificity of the RPA assays, they were evaluated with gDNA from *Ascaris lumbricoides* (AL), *Trichuris trichiura* (TT), *Wuchereria bancrofti* (WB), *Loa loa* (LL), *Onchocerca volvulus* (OV), and *Ss*). As shown in Figure 5, only gDNA from *Ss* and the synthetic oligo used as positive control were positive across all assays. No cross-amplification was observed with gDNA derived from any of the other helminth parasites demonstrating excellent target specificity.

### Performance of NIE RPA and NIE CRISPR-Cas12a in comparison with qPCR-positive patient samples.

**Figure 6.**
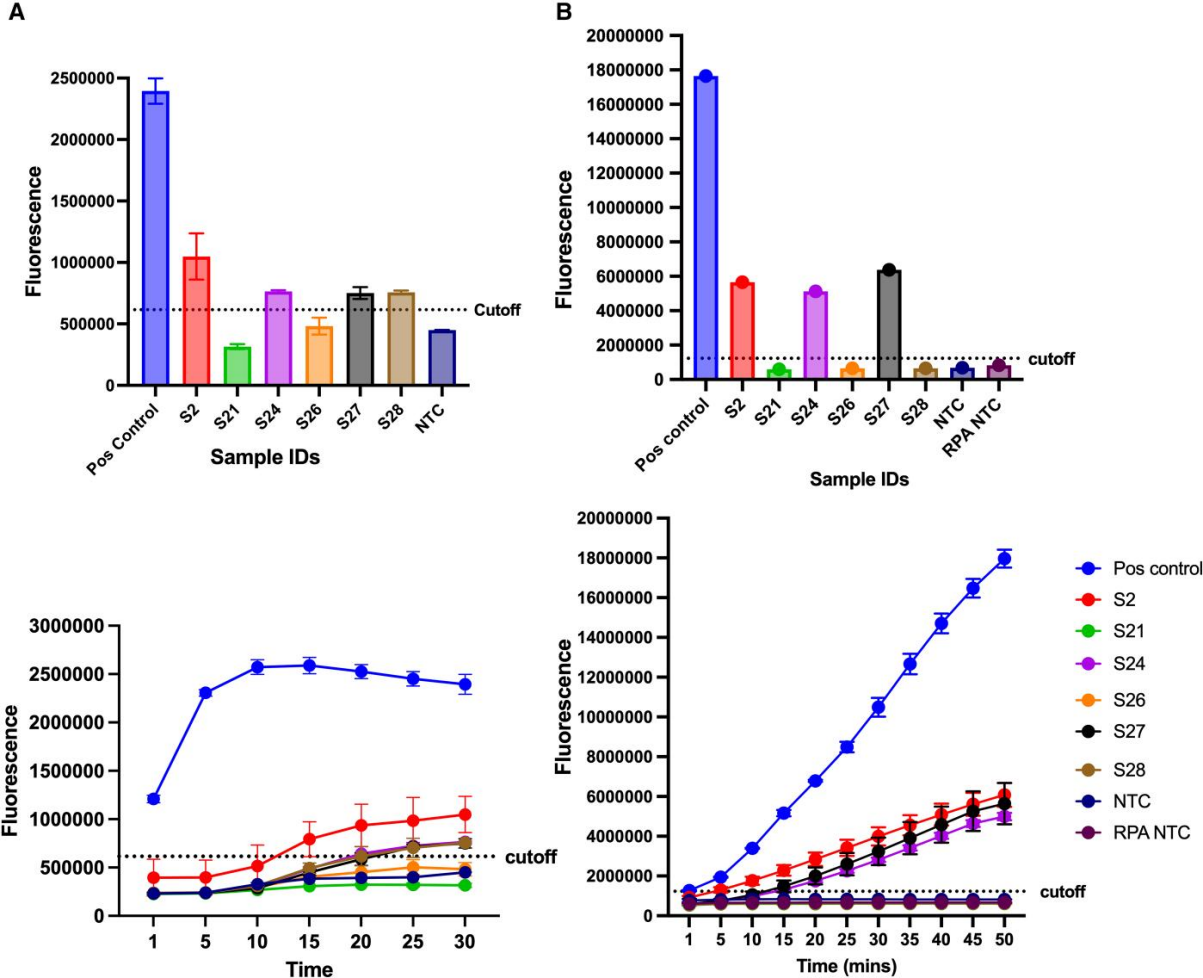
Performance of NIE assays on qPCR positive samples. Six samples known to be positive for *Ss* were tested on the NIE RPA (**A**) and RPA–CRISPR-Cas12a (**B**). Four samples out of six were positive with the NIE RPA and three with the RPA–CRISPR-Cas12a. Positive samples show fluorescence intensities above the cutoff line.

*S. stercoralis* qPCR-positive gDNA extracted from stool samples collected in an endemic area were tested on these assays in full reactions using 2 *µ*L gDNA as template. Only the NIE targeting assays were tested due to relatively higher LODs. A total of six *Ss* qPCR-positive samples were tested for comparative performance purposes. As illustrated in Figure 6, four of the six qPCR-positive samples tested positive with the NIE RPA and three with the less sensitive CRISPR-Cas12a. For most positive samples in our newly developed assays, positivity was found in samples with higher gDNA abundance with qPCR Ct ≤31. This finding is supported by the strong negative linear correlation between the assays with respective coefficients of *r = −*0.87 (*P* = 0.023) and *r = −*0.75 for NIE RPA and CRISPR-Cas12a (*P* = 0.053).

## DISCUSSION

Despite advances in serology and molecular detection of *Ss* infection, diagnosis is still challenging. Isothermal amplification techniques such as RPA and CRISPR-Cas12a systems are known for their rapidity and functionality for use in POC,^27^^,^^28^ as well as for their potential to surmount common issues such as the need for constant electricity and complex equipment, reducing infrastructure prerequisites.^29^^,^^30^ In this study, two EXO RPA assays—targeting the NIE antigen gene and the *Ss*-DRS—and one NIE RPA–CRISPR-Cas12a assay were successfully developed for detection of *Ss* DNA in stool. The NIE gene yielded a higher analytical sensitivity (LOD 1-100 fg) both for the EXO RPA and the RPA–CRISPR-Cas12a than the *Ss*-DRS with a LOD of 1 pg/*µ*L. Although our study highlights the need for further clinical analyses with larger clinical samples, these findings demonstrate the consistent and rapid detection of *Ss* DNA in stool for all three assays.

The NIE-targeting EXO RPA assay demonstrated superior analytical sensitivity, detecting 1 fg/*µ*L of genomic DNA. This performance aligns with the known diagnostic value of the NIE antigen, which has been extensively validated in serological assays due to high immunoreactivity and specificity for *Ss* infection. The NIE antigen has been used in several serological diagnostic tests showing high sensitivities and low rates of false positives.^10^^,^^12^^,^^31^^–^^34^ Regardless, the good performance of RPAs has been attributed to high resistance to PCR inhibitors.^35^ To the best of our knowledge, this is the first time the NIE gene has been exploited for the molecular diagnosis of *Ss*. Although the utility of *Ss*-DRS in molecular diagnosis^17^^,^^18^^,^^36^ is documented, NIE has primarily been the focus of serological immunoassays.^10^^,^^12^^,^^13^ It was interesting that *Ss*-DRS, a dispersed repetitive sequence, does not have the same sensitivity as *Ss*-NIE, which is a single-copy gene that maps to chromosome 2 of the *Ss* genome (PRJNA930454). The probability of promiscuous NIE primers/probe binding in multiple regions of the genome was excluded because the primers/probe used in the assay were highly specific to this region (chromosome 2—location from the primer analyses). Because the *Ss*-DRS oligo used as a positive control had no issues, we can speculate that the *Ss*-DRS repetitive region probably lies in highly dense heterochromatin that is probably not readily accessible and thus have a lower threshold. The ability to amplify this locus at the femtogram level makes this assay a potential tool for detecting low parasite burdens typically observed in chronic strongyloidiasis, where larval output is scanty and highly variable.^37^

The *Ss*-DRS-targeting EXO RPA assay demonstrated lower but still high analytical sensitivity, with reliable detection to 1 pg/*µ*L. Comparatively, the LOD reported herein is lower, in comparison with previously developed qPCR targeting the *Ss* repeat sequence with a consistent detection of gDNA at concentrations down to 1 fg/*µ*L.^18^ Repetitive loci can be strong targets for amplification-based diagnostics, but they also present challenges in primer design due to potential intra-repeat variability, affecting primer binding affinity^38^ or presence of secondary structures. Duncan et al.^39^ developed a pan-*Leishmania* qPCR targeting the tandemly repetitive sequence for DNA amplification but discovered lower sensitivity in two species that could be attributed to the imperfect nature or evolutionary pressure exerted on tandem repeats or still a relatively reduced number of repeats in those species. In all, both of our newly developed EXO RPAs (NIE and *Ss*-DRS) outperformed the only available *Ss* RPA-lateral flow test targeting the 18s rRNA gene^23^ with a LOD of 20 pg/*µ*L.

The RPA–CRISPR-Cas12a assay targeting NIE demonstrated an LOD of 500 fg/*µ*L. Although a little less sensitive than the corresponding EXO RPA, CRISPR-Cas12a systems perform optimally when combined with RPA and have the advantage of an improved specificity through a secondary recognition system with the likelihood of reducing false positives that may arise from RPA alone.^35^^,^^40^ This is the first time RPA is being coupled with CRISPR-Cas12a for *Ss* detection. These findings are consistent with existing literature using CRISPR-based assays for pathogen detection. Besides, similar findings were demonstrated by Lei et al.,^25^ with a LOD of 3.3 genomic DNA copies/*µ*L in a RPA–CRISPR-Cas12a–based assay for the detection of environmental *Toxoplasma gondii* and Shen et al.^41^ with detection limit of 1 gDNA copy/*µ*L for *Brucella melitensis* detection.

The specificities of all three assays were confirmed by testing against a panel of gDNA derived from other soil transmitted helminths and filarial parasites. The results indicated that only the *Ss*-positive samples produced a strong fluorescent signal, whereas no signal was observed for the other parasites. The absence of cross-reactivity with other parasites highlights the robustness of our assays and their potential for accurate diagnosis in both clinical and field settings.

When tested with six qPCR-positive cryopreserved stool samples, NIE EXO RPA detected *Ss* in four of six samples, whereas CRISPR-Cas12a detected three with both assays showing correlations between lower Ct values in qPCR and higher fluorescence intensity RPA. Though our study is based on a small number of clinical samples, these findings highlight that amplification at clinically relevant parasite loads is achievable. The slightly lower performance of the CRISPR-Cas12a assay may simply reflect differences in amplification kinetics between assays. Again, the EXO RPAs can be completed in just 30 minutes and 1 hour for NIE RPA–CRISPR-Cas12a tailoring them to perfectly fit the demands of both clinical and field environments. These assays address the several gaps observed in the diagnosis of *Ss* using serology and qPCR. Moreover, RPAs can rely on the human body temperature as a source of energy during incubation and can actually be done at ambient temperature. Sears et al.^30^ obtained the same LODs when using a heat block, shirt pocket, and thermal cycler.

## CONCLUSION

In conclusion, the *Ss*-NIE and *Ss*-DRS EXO RPAs, and NIE RPA–CRISPR-Cas12a assays developed in this study put forth three rapid and field-deployable molecular detection tools that maintain high analytical performance while minimizing equipment requirements, suitable for low-resource areas in endemic regions and screening of patients at the clinical level.

## Supplemental Materials

10.4269/ajtmh.26-0042 Supplemental Materials
