# Supplementary material for: Development of Recombinase Polymerase Amplification and CRISPR-Cas12a–Enhanced Isothermal Amplification Assays for Strongyloides stercoralis DNA Detection: A Pilot Study
Source: Am J Trop Med Hyg. 2026 Apr 21;114(6):1157–64. doi: 10.4269/ajtmh.26-0042 (PMC13235594; doi:10.4269/ajtmh.26-0042)
Supplement: Supplemental Materials [file tpmd260042.SD1.pdf]

Supplementary Materials

S1 Table: RPA forward and reverse primers sequences tested

Assay Design	Forward primer	Reverse primer
Ss-NIE EXO RPA	GCTCAAGCTTATGCTGAAGTAATTGCTAG	GTAAAATGTCCAGTTGCAGGACTAAATCCAGG
		TGTGTAAAATGTCCAGTTGCAGGACTAAATCC
		CCAGCATGAGTTGTACCTTTCCATACTAATTG
Ss-DRS EXO RPA	ACGCTCCAGAATTAGTTCCAGTTGAATAACAG	TTGTGAGGGGTTGGAAACTGTTGCTTTACTGG
		GGTTGGAAACTGTTGCTTTACTGGAGCT
	CTCACGCTCCAGAATTAGTTCCAGTTGAATAA	TTGTGAGGGGTTGGAAACTGTTGCTTTA
		CTCTTTTGTGAGGGGTTGGAAACTGTTG
Ss-NIE basic RPA	ATCGAATTGAGGGAACAGGAGAAAATCTTGCT	CACCAAATCCAGCATGAGTTGTACCTTTCCAT
	CGAATTGAGGGAACAGGAGAAAATCTTGC	ACACCAAATCCAGCATGAGTTGTACCTTTCCA

S2 Table: Guide RNA sequences tested on CRISPR/Cas12a system

**Guide RNA**	**Sequence**
crRNA 294	TTTA GCACCATGAGCTATTCTATAT
crRNA 489	TTTA AAATTATATAAAGCTATTTCA
crRNA 548	TTTA CACAATTAGTATGGAAAGGTA
crRNA 587	TTTG GTGTTGTTGAAAAAGGGGATA

Supplementary Data: Oligo Sequences

NIE EXO RPA oligo

AGATTAGAACATGATCCAAAAAATCGAATTGAGGGAACAGGAGAAAATCTTGCTTAT
GGAACAACATTTATTGGCCATTTAGCTGTAAAAGGATGGTATGATGAAATAGCTTTATA
TAATTTTAAAAAACCTGGATTTAGTCCTGCAACTGGACATTTTACACAATTAGTATGGA
AAGGTACAACTCATGCTGGATTTGG

NIE basic RPA oligo

AAGCTCAAGCTTATGCTGAAGTAATTGCTAGATTAGGAAGATTAGAACATGATCCAAA
AAATCGAATTGAGGGAACAGGAGAAAATCTTGCTTATGGAACAACATTTATTGGCCAT
TTAGCTGTAAAAGGATGGTATGATGAAATAGCTTTATATAATTTTAAAAAACCTGGATT
TAGTCCTGCAACTGGACATTTTACA

Ss-DRS EXO RPA oligo

TCTCACGCTCCAGAATTAGTTCCAGTTGAATAACAGTCTCCAGTTCACTCCAGAAGAG
TTCCTATAATCCTAACTCAGCTCCAGTAAAGCAACAGTTTCCAACCCCTCACAAAAGA
GCTTCTATGCTTTCGACTAAGCTGCAGTAT

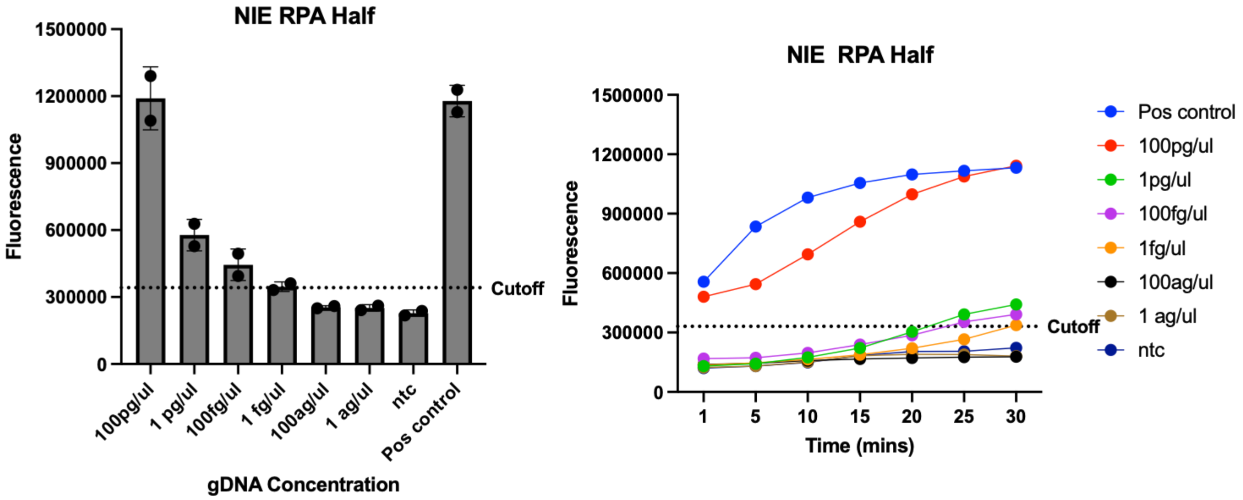

S1 Figure: Limits of detection of NIE RPA assay with half reactions showing a limit of detection of 100fg/ μL.
